# Draft Genome Sequence of *Alcanivorax* sp. Strain KX64203 Isolated from Deep-Sea Sediments of Iheya North, Okinawa Trough

**DOI:** 10.1128/genomeA.00872-16

**Published:** 2016-08-25

**Authors:** Huan Zhang, Rui Liu, Mengqiang Wang, Hao Wang, Qiang Gao, Zhanhui Hou, Dahai Gao, Lingling Wang

**Affiliations:** a Key Laboratory of Experimental Marine Biology, Institute of Oceanology, Chinese Academy of Sciences, Qingdao, China; b Key Laboratory of Mariculture & Stock Enhancement in North China's Sea, Ministry of Agriculture, Dalian Ocean University, Dalian, China

## Abstract

This report describes the draft genome sequence of *Alcanivorax* sp. strain KX64203, isolated from deep-sea sediment samples. The reads generated by an Ion Torrent PGM were assembled into contigs, with a total size of 4.76 Mb. The data will improve our understanding of the strain’s function in alkane degradation.

## GENOME ANNOUNCEMENT

Bacteria of the genus *Alcanivorax* belong to a group of slow-growing marine hydrocarbonoclastic bacteria that can use oil hydrocarbons as its exclusive source of carbon and energy (1). By now, the genome sequences of six species in this genus isolated from various marine environments have been reported, including *A. borkumensis* (1), *A. pacificus* W11-5^T^ (2), *A. hongdengensis* A-11-3^T^ (3), *A. dieselolei* B5^T^ (4), and *A. jadensis*. The *Alcanivorax* sp. strain KX64203 was isolated from sediment samples which were collected by using an electrohydraulic grab with an underwater television camera during the cruise conducted by the scientific research vessel KEXUE in Okinawa Trough (126°54.32′ E, 27°48.47′ N, ~1190-m depth, around a hydrothermal vent). Analysis of the 16S rRNA gene sequence (GenBank accession number KU954765) showed that it shared 99% identity (100% coverage) with that of *A. dieselolei* strain PTG4-3, and almost 99% identity with this gene in other *Alcanivorax* strains.

The sequencing reads were generated by an Ion Torrent PGM using 314 Chip version 2 and a 400-bp sequencing kit, assembled by the Torrent SPAdes plugin (version 2.3) *de novo* assembler, and then merged using a CISA contig integrator (5). Protein-coding sequences were predicted by Glimmer software (version 3.02) (6) Ribosomal RNA genes were detected using RNAmmer software version 1.2 (7) and tRNA genes were detected using tRNAscan-SE (8).

The genome of KX64203 consisted of 4,757,396 bases in 79 contigs (*N*_50_ 159,927 bp and *N*_90_ 36,849 bp), and had a G+C content of 61.4%. Both the genome size and G+C content were similar to published data for *A. dieselolei* type strain B5 (total length of 4.86 Mb and G+C% of 61.5%). Forty-three tRNA genes for all 20 amino acids and six rRNA genes were identified in the draft genome sequence. There were a total of 4,464 putative open reading frames (with an average size of 932 bp), giving a coding intensity of 87.43%. A total of 2,988 proteins were assigned to cluster of orthologous group (COG) families. The genes encoding three integral-membrane alkane monooxygenases (AlkB) and only one cytochrome P450 enzyme were found in the genome. Moreover, there were also some other proteins involved in the alkane degradation pathway, including three AlkKs, one AlkL, and one AlkN. Two genes, rubA and rubB encoding a rubredoxin and a rubredoxin reductase, respectively, were likely to be involved in alkane catabolism. The genome sequence and its gene annotation shed new light on the functional genomics of this species.

### Accession number(s).

This whole-genome shotgun project has been deposited in DDBJ/ENA/GenBank under the accession no. LVIC00000000. The version described in this study is the first version LVIC01000000.
